# Huang Lian for ulcerative colitis: A protocol of systematic review and meta-analysis of randomized clinical trials

**DOI:** 10.1097/MD.0000000000022457

**Published:** 2020-10-02

**Authors:** Tinglin Li, Mengzhu Wu, Lizhen Wang, Xiaohan Song, Peimin Feng

**Affiliations:** a Hospital of Chengdu University of Traditional Chinese Medicine; b Chengdu University of Traditional Chinese Medicine, Chengdu, Sichuan, China.

**Keywords:** Huanglian (Rhizoma coptidis), UC, meta-analysis and systematic review, protocol

## Abstract

**Introduction:**

Ulcerative Colitis is a chronic nonspecific inflammatory disease of the colon and rectum, which is of global concern. It has the characteristics of a long course of disease and repeated attacks, which seriously affects the quality of life and economic and social development of the affected population. The treatment of UC by herb Huanglian and compound prescription contain Huanglian have been proved. However, due to the lack of evidence, there is no specific method or suggestion, it is necessary to systematically evaluate coptidis so as to provide effective evidence for further research.

**Methods and analysis:**

The following databases will be searched from their inception to June 2020: Electronic databases included PubMed, Embase, Cochrane Library, Web of Science, Nature, Science Online, WanFang China Biomedical Database, VIP Medical Information, CNKI (Knowledge Infrastructure of China). Main outcomes: Colonoscopy, improved condition (tenesmus), stool routine. Additional outcomes: Electrocardiogram (ECG), erythrocyte (RBC), leukocyte (WBC), platelet (PLT). Data will be extracted by 2 researchers independently, risk of bias of the meta-analysis will be evaluated based on the Cochrane Handbook for Systematic Reviews of Interventions. All data analysis will be conducted by data statistics software Review Manager V.5.3. and Stata V.12.0.

**Results:**

The results of this study will systematically evaluate the efficacy and safety of rhizoma coptidis intervention in patients with Ulcerative Colitis.

**Conclusion:**

Through the systematic review of this study, the published evidence of rhizoma coptidis on the treatment of UC is summarized so as to further guide its promotion and application.

**Ethics and communication:**

This study is a systematic review, the outcomes are based on the published evidence, so examination and agreement by the ethics committee are not required in this study. We intend to publish the study results in a journal or conference presentations.

**Open Science Fra Mework (OSF) Registration Number:**

August 27, 2020. osf.io/7nh3k (https://osf.io/7nh3k).

## 1 Introduction

Ulcerative Colitis (UC) is a chronic nonspecific inflammatory disease involving the rectum and colon, with abdominal pain, diarrhea, mucous pus and blood stool, and tenesmus as the main clinical manifestations.^[1]^ Studies have found that with the changes in lifestyle and diet structure, the incidence of Ulcerative Colitis is on the rise year by year. In Asia, the annual incidence of UC is 1.0/100,000–2.0/100,000.^[2]^In the treatment, amino salicylic acid,^[3]^ glucocorticoids^[4]^ and other drugs are often used in modern medicine for symptomatic treatment, but there are many problems, such as low overall cure rate, large adverse reactions, easy relapse after drug withdrawal, long-term drug dependence, and long treatment cycle.^[5]^

In recent years, Traditional Chinese medicine has been widely used in animal experimental studies of UC, and its effectiveness has been fully proved.^[6]^ A number of modern clinical studies have shown that herb Huanglian and compound prescription contain Huanglian have played a significant role in the treatment of UC at all stages,^[7]^ but no clear conclusion has been drawn on their efficacy and safety. Therefore, this study intends to use the systematic evaluation and meta-analysis methods of Huanglian or compound prescription contain Huanglian in the treatment of Ulcerative Colitis to evaluate its efficacy and safety.

## 2 Methods

### 2.1 Study registration

The protocol is pre-registered with the OSF (Open Science Framework). August 27, 2020.osf.io/7nh3k(https://osf.io/7nh3k). The protocol will follow the statement guidelines of Preferred Reporting Items for Systematic Reviews and Meta-Analyses Protocols (PRISMAP),^[8]^ Changes will be reported in the full review as required.

### 2.2 Inclusion and exclusion criteria for study selection

#### 2.2.1 Inclusion criteria

The inclusion criteria were all randomized controlled trials (RCTS), and Huanglian or compound prescription contain Huanglian was mainly used for the treatment of UC. The test language includes only Chinese or English.

#### 2.2.2 Exclusion criteria

The following courses will be excluded

1. Age under 18
2. Severe allergic constitution and allergy to Huanglian, compound prescription contain Huanglian and its compound.
3. Non - RCTs and Quasi - RCTs
4. Those with particularly serious medical conditions requiring emergency treatment
5. Case series and Reviews
6. Animal research.

### 2.3 Types of participants

The type of subjects included patients diagnosed with Ulcerative Colitis, regardless of degree, and possible complications. All patients should be treated with Huanglian herbs, or with Huanglian mainly combined with other conventional treatment methods. There is no sense of gender, race, or education.

### 2.4 Experimental interventions

Huanglian or compound prescription contain Huanglian should be the main treatment.

### 2.5 Control interventions

Intervention measures include: no treatment, placebo, non-drug intervention (such as diet, exercise, etc.), conventional western medicine (such as mesalazine, etc.), and retention enema. Joint interventions are allowed as long as all groups in a randomized trial receive the same joint intervention.

### 2.6 Types of measurement results

#### 2.6.1 Main results

1. Colonoscopy;
2. Improvement of tenesmus;
3. Stool routine.

#### 2.6.2 Additional outcomes

1. Electrocardiogram (ECG);
2. Red blood cells (RBC);
3. White blood cells (WBC);
4. Platelet (PLT).

## 3 Data source

### 3.1 Electronic search

The following data bases will be searched to identify eligible studies: PubMed, Embase, Cochrane Library, Web of Science, Nature, Science on line, Chinese Biomedical Database WanFang, VIP medicine information, and CNKI (China National Knowledge Infrastructure). The time range is: the starting time is determined according to the first literature available, and the deadline is August 2020.

### 3.2 Other search resources

**Figure 1:**
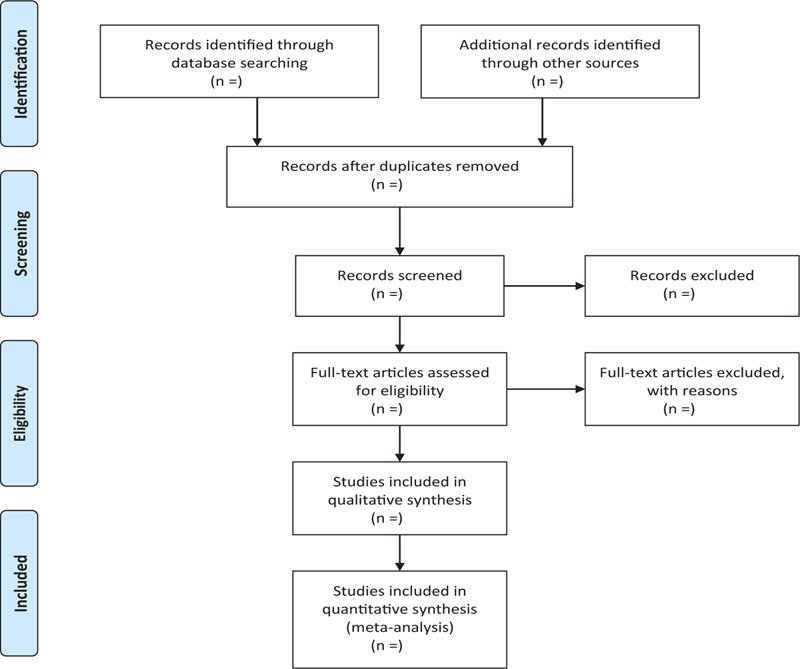
The research flowchart. This figure shows the identification, screening, eligibility and included when we searching articles.

In order to get more complete evidence, we will also retrieve other related documents by manually, such as medical textbooks, clinical laboratory manuals and so on. If it is necessary we will contact with trail author to obtain the latest clinical data. Moreover, studies associated with the review will be identified via evaluating related conference proceedings. The research flowchart is shown in Figure 1.

### 3.3 Search strategy

The following search terms will be used: randomized controlled trials/RCTS; Ulcerative Colitis/UC; traditional chinese medicine/TCM; huang-lian/Huanglian/coptis. Different retrieval strategies in Chinese and foreign databases will be used. Language restrictions are Chinese and English. There is no publication restriction. Here we take the search strategy in PubMed as an example and list in Table 1.

**Table 1:**
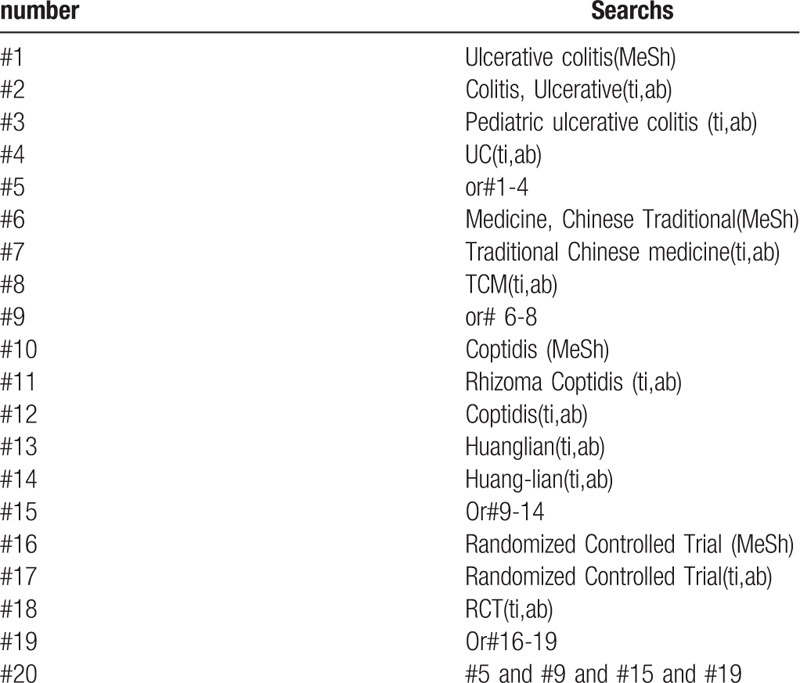
Search stragtegy sample of PubMed.

## 4 Data collection and analysis

### 4.1 Research selection

All articles in the search results were independently evaluated by 2 independent researchers (TL, MW) based on inclusion criteria and exclusion criteria. The evaluator will then independently extract and collect the data included in the study using a pre-designed data collection form. Differences will be discussed and resolved by consensus by corresponding authors (PF).

### 4.2 Data extraction and management

The following information will be extracted from each study:

Normal test features: title, author, year.

Baseline data: sample size, age, gender, diagnostic criteria, and course of disease.

Intervention: control and intervention of Huang Lian dosage and intervention details.

If the information is insufficient, we will contact experts and authors in the field for relevant information.

### 4.3 Assess report quality and risk of bias

The risk of bias will be assessed by two independent authors (TL and MW), together with completing the STRICTA checklist.^[9]^ The Cochrane System Evaluators Manual give the evaluation criteria for authors to evaluated the RCTs quality. Assessing the risk of bias:

1. Random sequence generation;
2. Allocate and hide;
3. Blind participants and personnel;
4. Blindness in result evaluation;
5. Incomplete result data;
6. Selective reporting of results;
7. Other biases.

Any objections will be discussed or consulted by a third reviewer. Each will be described from 3 levels of “high risk”, “low risk” and “unclear”.

### 4.4 A measure of therapeutic effectiveness

Dichotomous results will be represented by odds ratios (ORs), while continuous data will be represented by standardized mean differences (SMD). All of these results report 95% CI.

### 4.5 Missing data management

We will obtain the missing data by contacting the corresponding author. If there is no response, we will only analyze the existing data and describe the causes and effects of this exclusion in the paper.

### 4.6 Evaluation report deviation

Publication bias will be explored through funnel plot analysis. If the funnel graph is asymmetric, it will be assessed through Egger and Beg inspection, and *P* value <.05 indicates significant publication bias.

### 4.7 Evaluation of heterogeneity

There are mainly 2 methods to test heterogeneity, namely graphical method (funnel plot, forest plot) and statistical test (Q value statistical test, *I* ^2^ statistical test, H statistical test). *I* ^2^ statistical test showed that when *I* ^2^ was 0, it indicated complete homogeneity of the study; if *I* ^2^> was 50%, it indicated heterogeneity of the study.

### 4.8 Data synthesis and grading of evidence quality

The results will be analyzed using RevMan 5.0 software provided by the Cochrane Collaboration on Network. Binary data are represented by odds ratios and continuous data by mean difference (MD). In order to test the heterogeneity of study results, when *I* < 50% or *P* > .1, the heterogeneity was significant. The random-effect model was used in the meta-analysis; otherwise, the fixed-effect model was selected.

### 4.9 Subgroup analysis

#### 4.9.1 Sensitivity analysis

Sensitivity analysis can not only assess the stability and reliability of the conclusions of meta-analysis, but also assess whether the changes in the results are related to the influence of a single study. If the stability of the conclusion is poor, we can realize that when the heterogeneity test result is heterogeneous, we need to clarify the source of heterogeneity through subgroup analysis. Analysis of design, severity of the disease, age, gender, mild, and severe Ulcerative Colitis treatment effect. We will also remove studies of low and/or medium quality to check the robustness of the results.

Improve stability by changing analysis models, inclusion and exclusion criteria, or by excluding a certain type of literature.

### 4.10 Morality and communication

We will publish systematic review results in peer-reviewed journals and disseminate them at conferences or in peer-reviewed publications. Aggregated published data will be used to cull personal data without the need for ethical approval or informed patient consent.

## 5 Discussion

Huang Lian is a traditional Chinese medicine for clearing away heat and detoxifying. Can be used for a variety of inflammatory diseases, such as acute pancreatitis, pneumonia, Ulcerative Colitis, etc. Its treatment of Ulcerative Colitis is mainly reflected in the following aspects:

1. By activating the expression of PPARγ gene and then inhibiting the activation of p38MAPK, it alleviates the inflammatory damage of colon tissue in UC rats^[10]^;
2. Maintain the homeostasis of the mechanical barrier of intestinal mucosa by inhibiting the destruction of intestinal stem cells and tight junction proteins^[11]^;
3. The symptoms of pus and blood stool and tenesmus were relieved faster^[12]^;
4. Reducing the inflammatory response in the intestine by reducing NF-kB expression and inhibiting the inflammatory response in the colon of UC.^[10]^

In addition, clinical trials have also confirmed that the traditional Chinese prescription containing Huang Lian, such as Huang Lian decoction, can significantly improve the clinical symptoms, biochemical examination and pathological biopsy indicators of patients with Ulcerative Colitis,^[13]^ and improve the quality of life of patients.

The effective effect of Huang Lian on Ulcerative Colitis may be related to its main active components.

Huang Lian mainly includes berberine and other bioactive alkaloids. Pharmacological function of berberine is the major component, and its resistance to ulcerative colitis lies mainly in the following characteristics:

1. the berberine can promote the recovery of ulcerative colitis in mice induced by DSS, inhibiting inflammatory response of macrophages and colon epithelial cells, promote macrophage apoptosis of colon, and can inhibit the activation of the NF-κB and MAPK pathway, cut DSS induced TNF, IFN-γ,KC, IL-17, to cut macrophages and colon epithelial cells in the production of proinflammatory cytokines, reduce fossae injury, and severe inflammatory lesion.^[14]^
2. Berberine can reduce the expression of pro-inflammatory cytokines IL-1, IL-6 and TNF- mRNA and CD68(markers of macrophages) in MICE with DSS-colonitis.^[15]^
3. Berberine reduces the expression of IFN-γand TNF-αby inhibiting the MLCK-MLC phosphorylation signaling pathway^[16]^;
4. berberine can improve proinflammatory cytokines in vitro induced by endoplasmic reticulum stress effect, to reduce the inflammatory reaction.^[17]^

But there are also research shows that the berberine in Huang Lian alkaloid is one of the most important toxic ingredients, clinical and animal experiments on its mortality and morbidity is not yet clear, the toxicological mechanism still needs further research.

To sum up, systematic review and meta-analysis are helpful to determine the potential value of Huang Lian. Huang Lian extract and its combination in the treatment of Ulcerative Colitis. Improve the quality of life of critically ill patients. This study can not only provide the basis for the release of guidelines for the treatment of Ulcerative Colitis, but also promote the application of Traditional Chinese medicine prescription, so that more patients can benefit.

## Author contributions

**Conceptualization:** Tinglin Li, Mengzhu Wu, Xiaohan Song.

**Data curation:** Tinglin Li, Peimin Feng, Lizhen Wang.

**Formal analysis:** Tinglin Li, Mengzhu Wu, Xiaohan Song.

**Funding acquisition:** Peimin Feng.

**Methodology:** Mengzhu Wu, Peimin Feng.

**Project administration:** Tinglin Li, Mengzhu Wu, Xiaohan Song.

**Resources:** Mengzhu Wu, Xiaohan Song, Lizhen Wang.

**Software:** Tinglin Li, Peimin Feng.

**Supervision:** Peimin Feng.

**Visualization:** Lizhen Wang.

**Writing – original draft:** Tinglin Li, Xiaohan Song.

**Writing – review and editing:** Tinglin Li, Lizhen Wang.
